# ChatGPT's ability or prompt quality: what determines the success of generating multiple-choice questions

**DOI:** 10.1016/j.acpath.2024.100119

**Published:** 2024-05-21

**Authors:** Yavuz Selim Kıyak

**Affiliations:** Department of Medical Education and Informatics, Faculty of Medicine, Gazi University, Ankara, Turkey

**Keywords:** ChatGPT, Multiple-choice questions, Prompt engineering, Artificial intelligence, Automatic item generation

I have read with great interest the study by Ngo et al.^1^ titled “ChatGPT 3.5 fails to write appropriate multiple choice practice exam questions.” The study aimed to assess whether ChatGPT could generate scientifically appropriate multiple-choice questions (MCQs). There is an ongoing demand for high-quality MCQs, as traditional methods of item writing are notoriously time-consuming and labor-intensive. The authors’ exploration of artificial intelligence (AI) as a tool to alleviate this burden is timely, particularly because the study was conducted soon after the public release of this AI model. The authors' efforts in this regard are commendable.

The authors interpreted their findings as a significant deficiency in ChatGPT 3.5's ability to produce appropriate MCQs. However, these findings could also suggest that ChatGPT's perceived shortcomings may not be due to the AI model itself but rather due to the insufficiently detailed prompts used in the study: “Write 4 multiple choice questions with 4 answers, including explanations for both correct and incorrect answers.” As this prompt demonstrates, effective prompting strategies such as providing precise instructions, focusing on core concepts and keywords, and numbering instructions^2^ to ChatGPT were not employed. The simplicity and lack of detailed descriptions in the prompts failed to leverage ChatGPT's capabilities. This is understandable, given the limited information available on creating effective prompts at the time of the study.

However, it would be better if the study would have been acknowledged this limitation and included a discussion on possibility of which prompt design impacts output quality. In contrast, well-structured prompts, like those developed by Zuckerman et al.^3^ and Kıyak,^4^ show how detailed and tailored inputs can leverage ChatGPT's utility in automatic item generation, even for generating case-based MCQs. While I do not suggest that the outputs would be ready for use without expert review, these well-designed prompts provide a more detailed description or clever design, guiding the AI to generate more accurate questions.

In conclusion, Ngo et al.'s study is valuable for highlighting ChatGPT's limitations in generating MCQs and underscores the need for human oversight on using outputs of AI. It also indirectly shows the critical importance of prompt design in maximizing ChatGPT's potential. By employing more sophisticated and detailed prompts, we can likely achieve more acceptable MCQs, even with ChatGPT 3.5.
